# Healthcare Service Quality Evaluated Using the Degree of Satisfaction of Patients in Romanian Community Pharmacies

**DOI:** 10.3390/healthcare11233038

**Published:** 2023-11-25

**Authors:** Magdalena Bîrsan, Alin-Viorel Focșa, Ana Caterina Cristofor, Sadiye-Ioana Scripcariu, Paula Antonoaea, Robert-Alexandru Vlad, Nicoleta Todoran, Adriana Ciurba, Cristinel Ionel Stan, Manuela Maria Apostol, Catalina Daniela Stan

**Affiliations:** 1 Department of Drug Industry and Pharmaceutical Biotechnology, Faculty of Pharmacy, “Grigore T. Popa” University of Medicine and Pharmacy Iasi, Iasi 16 Universităţii Street, 700115 Iași, Romania; 2 Morphofunctional Department I, Faculty of Medicine, “Grigore T. Popa” University of Medicine and Pharmacy Iasi, 16 Universităţii Street, 700115 Iași, Romania; 3 Mother and Child Health Department, Faculty of Medicine, “Grigore T. Popa” University of Medicine and Pharmacy Iasi, 16 Universităţii Street, 700115 Iași, Romania; 4 Department of Pharmaceutical Technology and Cosmetology, Faculty of Pharmacy, “George Emil Palade” University of Medicine, Pharmacy, Science, and Technology, 38 Gheorghe Marinescu Street, 540142 Targu Mures, Romania; 5 Faculty of Psychology and Educational Sciences, “Al I Cuza” University, 3 Toma Cozma Street, 700554 Iasi, Romania

**Keywords:** pharmaceutical services, patient satisfaction, community pharmacy marketing, patient expectations in the pharmacy

## Abstract

Community pharmacy has evolved a lot in recent years in terms of pharmaceutical services and marketing policies applied in Romania. This study aimed to evaluate the degree of patient satisfaction in community pharmacies in Iași, Romania correlated with the frequency of returning to the pharmacy, level of education, gender, and stress level at the time when the pharmacist dispenses the medication. A total of 30 community pharmacies were involved, and in a period of three months, they issued questionnaires to patients. 722 patients responded, and to verify the first research hypothesis, the Pearson correlation was applied. Statistical analysis revealed that there is a negative, medium-level, and significant correlation between the level of satisfaction with pharmaceutical services and the frequency of visits to the pharmacy, r = −0.342, *p* < 0.0001. There is also a significant, negative correlation of low intensity between the level of satisfaction with pharmaceutical services and patient status, r = −0.202, *p* < 0.0001. The degree of patient satisfaction is influenced by the quality of the basic pharmaceutical service offered, by the frequency of visits to the pharmacy, by the level of stress, and by social class.

## 1. Introduction

Community pharmacies are, for most people, often the patient’s first contact with the health field, offering both advice on the correct use of medicines and advice on public health education [1,2]. These activities support the health systems, by ensuring the continuity of drug therapy, maintaining the health of the population, and respecting restrictions [3,4,5,6]. Patient-centered pharmaceutical services bring multiple benefits to the health system as a whole. The pharmacist contributes to the success of the drug therapy recommended by the doctor by correctly releasing the drugs and by advising regarding the method of administration, to obtain maximum benefits and minimize the associated risks [7]. There are enough arguments that demonstrate the important role of the community pharmacist in supporting the health system, having an ideal position for prevention, management of chronic conditions, timely identification of aggravations, and referral of the patient to the doctor [8]. The pharmacists in community pharmacies in Romania provide counseling services regarding prescription or OTC drugs and provide valuable health information, without these services being recognized or remunerated [9,10,11].

**Figure 1:**
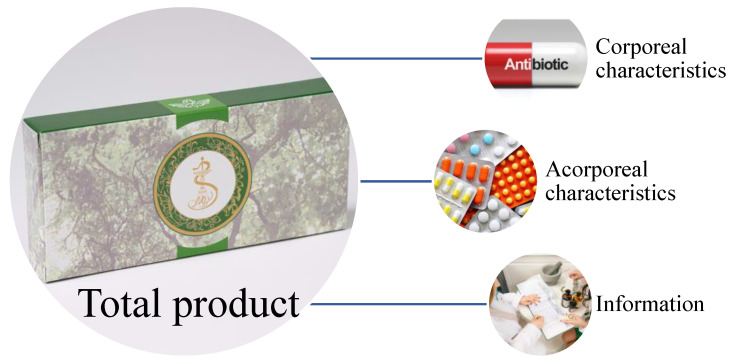
The characteristics of a total product sold in a community pharmacy.

According to the marketing definition, medicines are released from the pharmacy as a total product (Figure 1). The purchase of the total product implies the release of the three characteristics, corporeal characteristics, acorporeal characteristics, and information, which in the pharmaceutical field means patient counseling. Each characteristic of the product brings typical benefits. The pharmaceutical product cannot be sold with only one of the characteristics. They add up to the total product. The physical characteristics are those determined by the physical product such as its pharmaceutical form, the active substance, auxiliary substances, etc. The intangible characteristics are determined by those elements that add value to the product, such as the prospectus or its packaging. The strength of any medicine and the power of persuasion is offered by marketing and also by the information/counseling characteristics offered in the pharmacy to the patient [12]. 

Pharmacies have been privatized since 1991, and for more than 30 years different patient satisfaction policies have been used in the community pharmacies. The pharmaceutical market through privatization was relatively free, and at this moment the studies performed in Romania regarding the degree of satisfaction are very few. The general tendency, if we refer to the TV commercials, is to think that patients only want low prices for drugs [12,13,14,15,16].

The role of the pharmacist is extremely important for the effectiveness of the treatment because it can increase compliance with the treatment through the information provided. His role is not only to release the medicine but also to ensure that the information he transmits, once it is released, has been fully understood; that the treatment plan will be respected and monitored; and that in case of any unwanted or serious manifestations the patient contacts their doctor or pharmacist as soon as possible [17,18,19,20,21,22,23,24,25].

There are many theories about the degree of satisfaction of a consumer and the fulfillment criteria to achieve sales performance, but for this market segment, things are a little different. Today’s consumers demand variety and convenience. The correctly defined drug consumer, the community pharmacy patient, must be given extra attention because the pharmaceutical service is not just a commercial service, it is a wider service. Consumer satisfaction occurs when the customer is satisfied by a product/service that meets the consumer’s needs, wants, and expectations. To better understand consumer satisfaction, we need to look more fundamentally at specific satisfaction levels. We must also recognize that there are levels of consumer satisfaction that, in a sense, define the core ingredients and quality. 

All definitions for consumer satisfaction contain some common elements. When analyzed as a whole, three general components can be identified [26,27,28,29,30]. 

**Figure 2:**
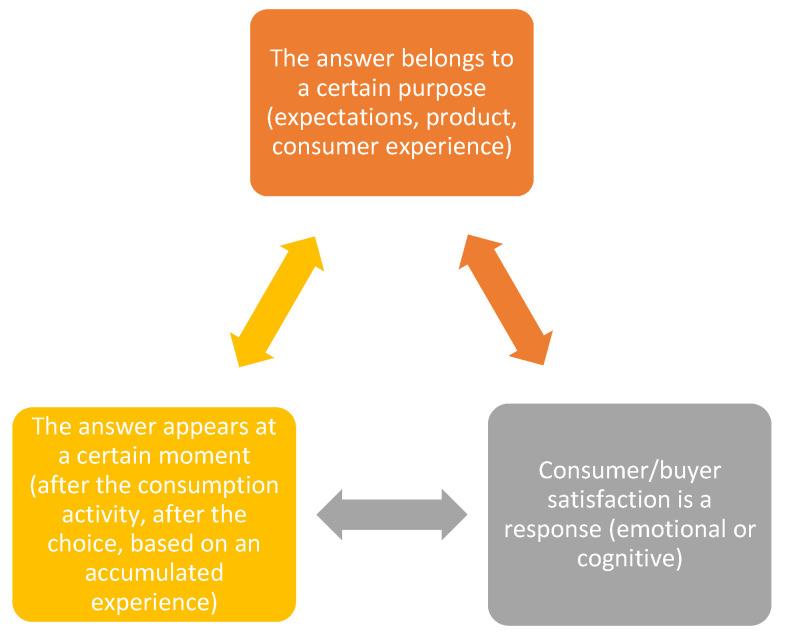
The most important theories regarding the degree of satisfaction of the consumer with the product/service.

In addition to the three theories presented in Figure 2, there are several classic tools and methodologies to identify and focus on customer satisfaction, including the Kano model. This type of model demonstrates that consumer satisfaction is closely related to the expectations of the final consumer. Consumer satisfaction is an indicator of loyalty to the product/service received. One of the hypotheses proposed in this study was the frequency of visits to the pharmacy, which constituted a predictive model for the level of patient satisfaction. Loyalty in this case was identified with the frequency of the patient returning to the community pharmacy.

### Alternatives to Patient Satisfaction 

Recognizing the limitations of measuring patient experience provided by satisfaction ratings, there are some recommendations to consider. Alternative and multi-method approaches to measuring patient experience could be based on expectations and preferences from the patient’s perspective in the context of the service received. More recent approaches have been proposed to assess experiential outcomes and the person-centeredness of health care interventions, such as consumer evaluation surveys of health care providers and systems, which are designed to collect information about the patient’s experience with a range of health care services at multiple levels of the health system [31,32,33,34]. 

Pharmaceutical services aimed at person-centered care are further emphasized in scientific frameworks where patient input and involvement can play an important role in understanding intervention feasibility, external forces, internal culture, and the ability to deliver services successfully [35,36,37]. However, the patient’s condition and vulnerability at the time of completing such a questionnaire should not be neglected. Sometimes a state of stress can influence an individual’s perception of the pharmaceutical service received.

The objective of our study was to research community pharmacies in Iași, Romania using the criteria by which patients choose the pharmacy or the pharmacist. The purpose of the study was to identify what the patients consider to be a priority to be satisfied. One of the criteria we wanted to check was the degree of satisfaction concerning the patient’s condition, education level, gender, or rural or urban origin. The degree of satisfaction evaluation was conducted by having patients from over 30 community pharmacies in Iași, Romania complete a questionnaire. The questionnaire contains questions to check regarding what the patient’s priority is when he is given medication, and what the criteria are for choosing the pharmacy where he receives his medication. One of the novelties of this study is that it not only identifies the criteria for choosing the pharmacy/pharmacist, but it also identifies whether a state of stress influences a patient’s degree of satisfaction.

The biggest goal of this research is to study the level of patient satisfaction with the services provided by pharmacists from community pharmacies and the correlation between the degree of satisfaction with pharmaceutical services and the level of stress perceived by the patient.

## 2. Materials and Methods

### 2.1. Study Design 

This is a cross-sectional study, in which the questionnaire method was used, collecting answers via items. The interpretation of the following aspects was made: the impact of kindness and communication ability of the pharmacist and the management policy used by the pharmacy to attract patients. The final questionnaire includes 18 items, is presented in annex 1, and covers aspects related to patient satisfaction with pharmaceutical services.

The first stage in the construction of the questionnaire involved establishing the dimensions we wanted to investigate with the help of the questionnaire. In this stage, the items for each dimension were defined. Taking into account the stages of construction and validation that the questionnaire goes through, assertions were formulated. In parallel with the formulation of the assertions, a record was formulated; a system for evaluating the statements was chosen; and, in accordance with it, a system for formulating the questions was chosen. For this questionnaire, the evaluation system we are considering is one using five steps: strongly disagree; disagree; neutral; agreement; and strong agreement.

The second stage consisted of the primary analysis of the intermediate form of the questionnaire. It followed the analysis of the initial questionnaire items within a group of experts made up of specialists: 6 pharmacists with over 15 years of experience, 2 university teaching staff from the Faculty of Pharmacy, a psychiatrist, and a psychologist with over 15 years of experience. Their purpose was to identify the extent to which the statements made refer to the purpose of the questionnaire. The conceptual theoretical framework and the dimensions targeted by the analysis were briefly explained to the experts. Analyzing the degree of agreement between the experts, some items from different categories were corrected. Content validity refers to the extent to which the assertions formulated by us target the content of the dimension we wish to investigate. 

In the third stage, we carried out its intermediate analysis. The questionnaire resulting from the evaluation within the expert group will be applied to a sample of subjects (patients), verifying through statistical analysis the internal consistency of the items and establishing the final form of the questionnaire (record, items, scoring). The intermediate analysis of the questionnaire will follow verification using statistical analysis of the “normality” of the distribution of the answers for each item and of the internal consistency of the items within each factor.

### 2.2. Study’s Hypothesis 

The study investigated the level of patient satisfaction with the services offered by the pharmacists from community pharmacies and the correlation between the degree of satisfaction with pharmaceutical services and the level of stress perceived by the patient. Four hypotheses were proposed.

**Hypothesis** **1** **(H1).** *The level of satisfaction with pharmaceutical services correlates with the variables of frequency of visits to the pharmacy, professional status, gender of subjects, and level of completed studies.*

**Hypothesis** **2** **(H2).** *The frequency of visits to the pharmacy will constitute a predictive model for the level of patient satisfaction.*

**Hypothesis** **3** **(H3).** *The frequency of visits to the pharmacy, the professional status, and the gender of the subjects will constitute a predictive model for the level of patient satisfaction.*

**Hypothesis** **4** **(H4).** *The level of stress perceived by the patient will influence the level of satisfaction with the pharmaceutical services received.*

### 2.3. Data Collection 

The study participants were randomly selected patients from the community pharmacies involved in the study who agreed to answer the proposed questionnaire. The selected participants were contacted by the pharmacist who dispensed their medication. The request was verbal together with informed consent that had to be completed in writing. The data were collected between March 2022 and May 2022. 

The questionnaires, which included both the 18 questions and an additional part, were completed in physical format by patients from the city of Iași who utilized the community pharmacies, and they completed it individually. The participants were informed about the confidentiality of the answers provided. They were also informed that they could stop filling out the questionnaire at any time and that the data obtained would only be used for research purposes. The 30 pharmacies involved in the studyemploy between 3 and 10 pharmacists per pharmacy. Even though there are over 210 pharmacists in the 30 pharmacies, we only reported on the pharmacists who interact with patients, namely those who sit at the counter. Only 156 pharmacists out of the 210 employees interacted with the patients involved in the study.

### 2.4. Research Ethics 

The research was carried out under the principles of pharmaceutical practice regulated in Romania and the Declaration of Helsinki (1975), as revised in 2000 [28]. The Ethics Committee has approved this study, number 3703/17.02.2023, with the assumption that responsibility to conduct the study follows the Research Law no. 206/27, updated in May 2004, as well as the European legislation in force.

### 2.5. Statistical Analysis

The data were analyzed using SPSS for Windows, Version 23.0 (SPSS Inc. Chicago, IL, USA). Pearson’s correlation, an independent sample t-test, and stepwise multiple regression analysis were used to analyze the results. Continuous and scale variables were presented, and the categorical ones were presented by frequencies and percentages (%).

One of the methods used to validate a questionnaire is the alpha-Cronbach internal consistency coefficient. An alpha-Cronbach internal consistency coefficient of at least 0.70 establishes that the instrument quantifies the dimension(s) the researcher set out to measure, as well as the respondents’ understanding of the meaning of the questionnaire’s statements.

## 3. Results

All patients who agreed to participate in the study completed the full questionnaire. There were no exclusion criteria. A total of 722 patients participated in our study, of which 67.31% were from an urban environment and 32.69% from a rural environment. A percentage of 43.83% had completed higher education, 18.86% were post-secondary graduates, 21.08% had a high school education, and only 16.23% had completed vocational school. Of the 722 patients, 55.26% were female patients, and 51.25% were employed. The distribution of patients according to the frequency of visits to the pharmacy is as follows: 20.76% go weekly, 33.24% use pharmaceutical services monthly, 24.38% every few months, and 21.61% annually.

The alpha-Cronbach coefficient of validity of the items for the group of patients participating in the study is 0.82.

The response percentage for women and men is understandable because the frequency of women is higher in pharmacies than that of men. It is well known that women take care of the whole family, including both children and the elderly, and the response rate of 55.26% confirmed this hypothesis. If we analyze the response rate of male patients, they come in less often to purchase drugs from the pharmacy, so the response rate of male patients was 44.74%.

From the data obtained, the frequency of people who buy products from the pharmacy is the highest among those with a university education (Figure 2). The next percentage, 21% of the 722 patients, are high school graduates. The level of education makes us aware of the importance of health.

### Participants Characteristics

All the patients who agreed to be involved in the study completed the questionnaire to the end, without any incomplete questions. The mean age of participants was 48 years. Most of the participants were female, over 55% (Table 1).

**Figure 3:**
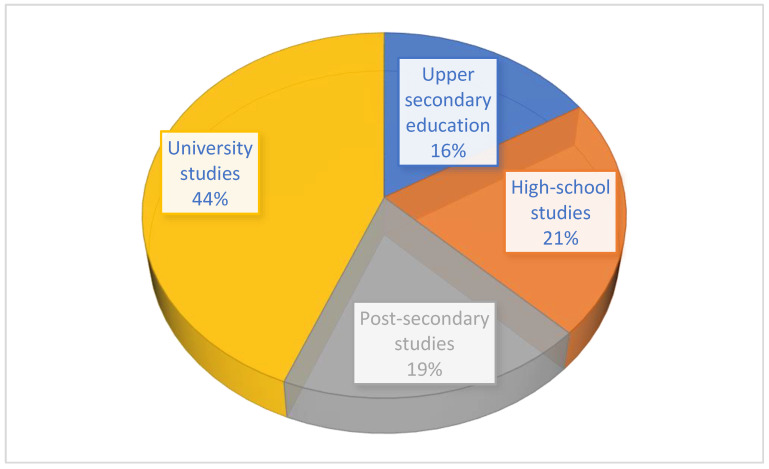
Education levels of patients involved in the study.

**Figure 4:**
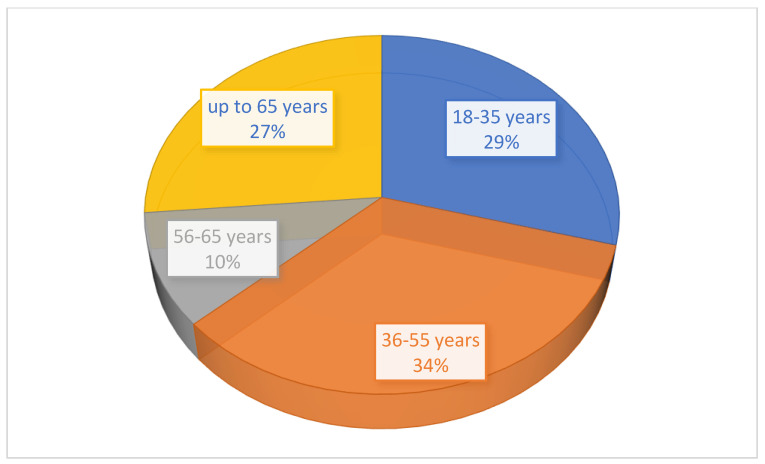
Age segments of patients involved in the study.

One of the hypotheses we wanted to check was the level of satisfaction according to certain parameters. The level of satisfaction with pharmaceutical services correlates with the variables: frequency of visits to the pharmacy, professional status, gender of the subjects, and the level of completed studies. To verify the first research hypothesis, the Pearson correlation was applied. Statistical analysis revealed that there is a negative, medium-level, and significant correlation between the level of satisfaction with pharmaceutical services and the frequency of visits to the pharmacy, r = −0.342, *p* < 0.0001. There is also a significant, negative correlation of low intensity between the level of satisfaction with pharmaceutical services and patient status, r = −0.202, *p* < 0.0001. There is a significant, negative, low-intensity relationship between the level of satisfaction with pharmaceutical services and patient status (r = −0.145, *p* < 0.0001), between the level of satisfaction with pharmaceutical services and patient gender (r = −0.126, *p* < 0.0001), and between the level of satisfaction with pharmaceutical services and the patient’s level of education (r = −0.111, *p* < 0.0001) (Figure 3 and Figure 4).

Among the 722 people involved in this study, most patients were between 36 and 55 years old (Figure 4). This high percentage is explainable because in this age range, there are people who have children or the elderly in their care, and, of course, they will buy medicines both for themselves and for the whole family, especially in the case of women. The lowest percentage was for people between 56 and 65 years old, when the state of health is not deeply affected, purchasing only treatments for themselves. After 65 years of age, health is more precarious, and implicitly they will need more extensive medication.

**Figure 5:**
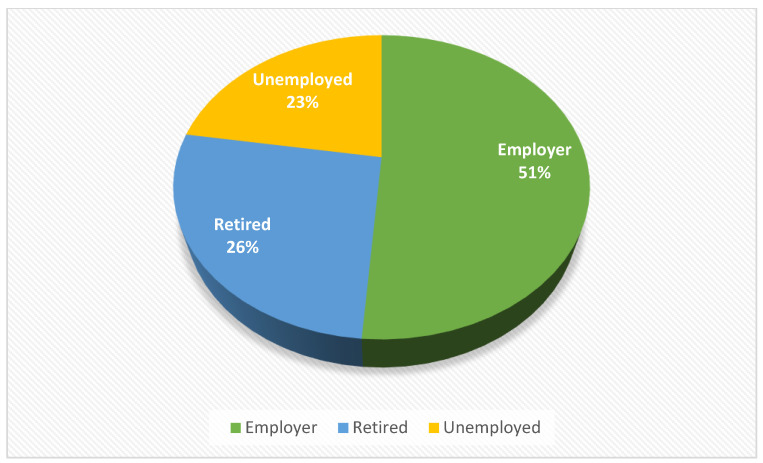
Social status of patients involved in the study.

Social status gave us very important information about the degree of satisfaction of patients in community pharmacies. The lowest percentage is occupied by patients who are not employed, which can be explained because a patient who is not insured will not be able to benefit from a medication on a compensated prescription (Figure 5). So, this type of patient will not be able to be issued RX-type drugs, only OTCs.

The frequency of visits to the pharmacy, the professional status, and the gender of the subjects constitute a predictive model for the level of patient satisfaction.

**Figure 6:**
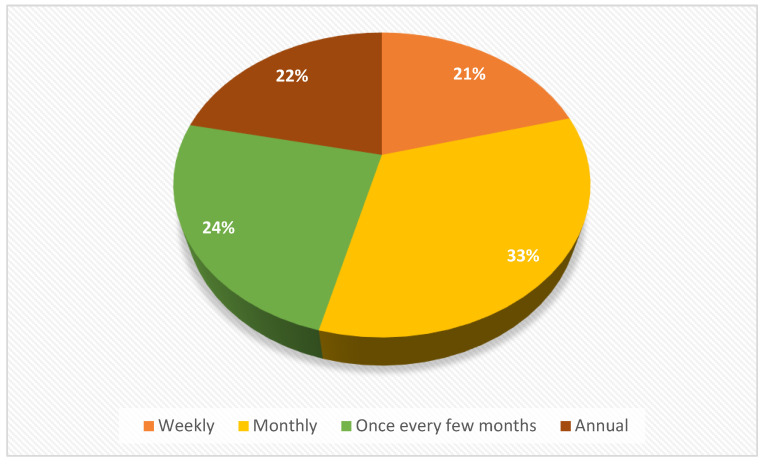
Frequency of visits to the pharmacy by the patients involved in the study.

Over 33% of all the patients who filled out the questionnaire responded that the frequency with which they go to the pharmacy for prescriptions or to buy OTCs or dietary supplements was monthly (Figure 6). That percentage can be explained by the monthly collaboration that chronic patients have with family doctors and their routine for receiving prescriptions. 

There is also a discussion about the 24% of people who purchase medicines from the pharmacy every few months. Many of the chronic prescriptions issued by family doctors are issued for three months, so patients consume products daily, but they only go to the pharmacy once every three months (Table 2).

**Figure 7:**
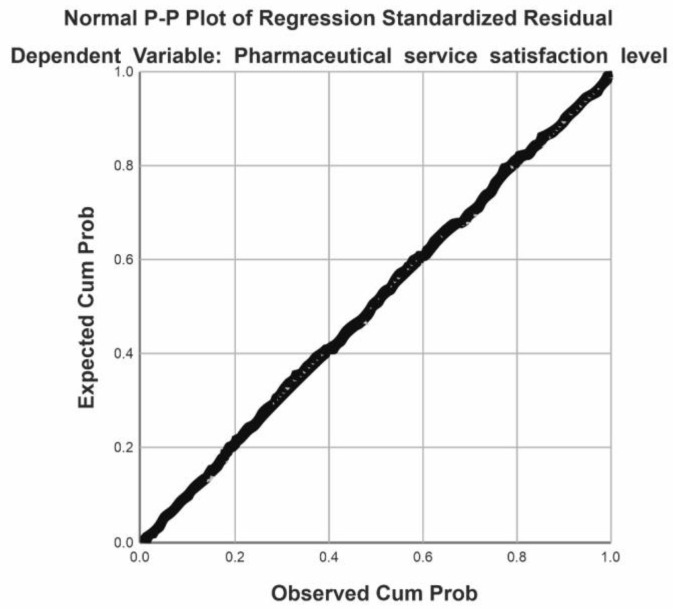
Graphic representation of the regression for the satisfaction variable towards pharmaceutical services, a predictor of the frequency of visits to the pharmacy.

Statistical correlations provide us with data on the degree of satisfaction of patients according to the frequency of visits and social status or professional level (Figure 7). Practically, the more often patients come to the pharmacy, the more satisfied they are with the pharmacist and the pharmaceutical services.

The frequency of visits to the pharmacy explains 11.7% of the variance of the variable satisfaction with pharmaceutical services (F (1,715) = 94.85, *p* < 0.0001). High levels of satisfaction with pharmaceutical services were associated with high numbers of visits to community pharmacies.

A percentage of 12.7% of the variance of the variable satisfaction with pharmaceutical services is explained by the variables’ frequency of visits to the pharmacy and patient status (F(1,7,14) = 7.85, *p* < 0.005). High levels of satisfaction with pharmaceutical services are associated with high levels of pharmacy visit frequency and patient status.

There is a significant, positive, low-intensity relationship between the level of stress perceived by the patient and the level of satisfaction with the pharmaceutical services received, r = 0.202, *p* < 0.0001. The level of statistics was also quantified depending on the patient’s state of stress. The general tendency is to believe that when we are stressed, we do not have the ability to receive advice about medication. In fact, the survey results demonstrate just the opposite. The degree of satisfaction is higher in the case of more stressed people; this result makes us think that pharmaceutical counseling does not provide the patient with more than information. Maybe this counseling regarding the patient’s understanding of the therapy and the treatment gives him confidence in the healthcare system, and, implicitly, the level of stress decreases, and the degree of satisfaction increases.

The application of the t-test for independent samples identified that patients participating in the study who have a high level of stress (M = 3.76, SD = 0.64) show a significantly higher high level of satisfaction with pharmaceutical services (t(708) = −3.25, *p* < 0.001) compared to patients who exhibited a low level of stress, (M = 3.61, SD = 0.61).

**Figure 8:**
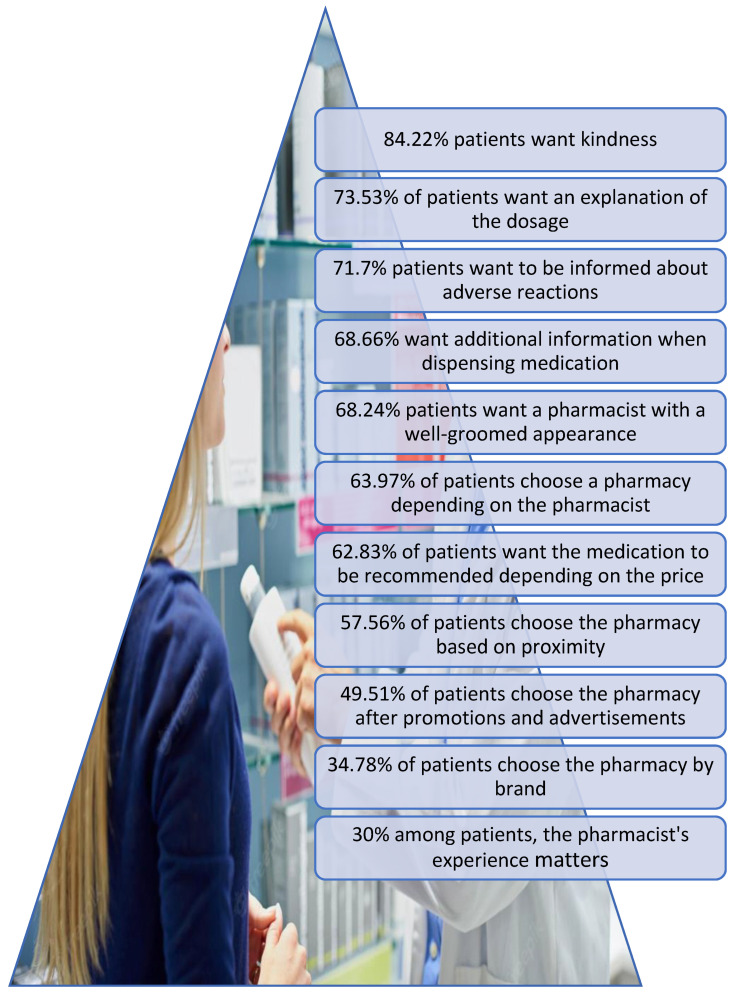
The most relevant results obtained from the questionnaire.

Analyzing the 18 questions answered by the patients, we realize that their degree of satisfaction depends on kindness, communication ability, and the pharmacist’s knowledge of the dosage and side effects of medicines (Figure 8). These three requirements were found in more than 70% of the 722 patients surveyed. To our surprise, neat dress and appearance are also extremely important for patients. From a psychological point of view, the patient considers the outfit a confidence factor. 

Although at this moment in the city of Iași, the chain pharmacies, which invest sufficient amounts of money in marketing, comprise over 60% of pharmacies, patients put more emphasis on choosing a pharmacy according to the friendliness, communication, and knowledge of the pharmacist than promotions and brand.

**Figure 9:**
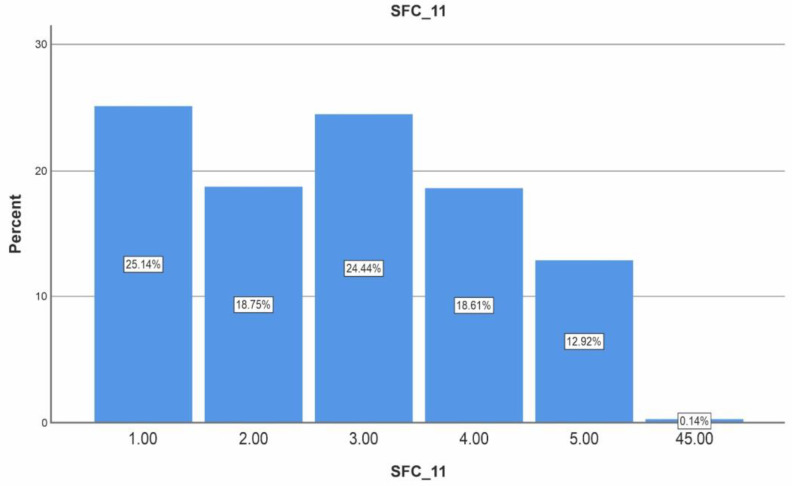
The results of the question “For me it is important that the pharmacist has at least 10 years of experience.” (1 strongly disagree, 2 disagree, 3 neutral, 4 agree, 5 strongly agree). SFC 11 means the abbreviation of the name of the specific questionnaire, satisfaction, question number 11.

As specialists in the field, we believe that the pharmacist’s experience is vital for communication, but the patient has a different view on this. Only for approximately 30% of the 722 patients surveyed, the pharmacist’s experience in the field matters (Figure 9).

The answers to question no. 11 of the questionnaire were among the most unexpected. Regarding the question ”For me, the pharmacist must have at least 10 years of experience” (1 strongly disagree, 2 disagree, 3 neutral, 4 agree, 5 strongly agree), only 31.53% of patients said experience of more than 10 years would influence their degree of patient satisfaction (Figure 8). This is a beneficial fact for this profession because young pharmacists can achieve maximum results if they show professionalism, good communication, and kindness to the patient.

## 4. Discussion

Our study wanted to identify and discover the situation in the city of Iasi, Romania, regarding patients’ level of satisfaction with the pharmaceutical services received in community pharmacies. A particularity of the study is the large number of patients participating in the study who graduated from higher education.

The 722 respondents from the community pharmacies in the city of Iasi, Romania provided an overview of the level of patient satisfaction in this area, especially regarding their perception of the pharmaceutical services received. The patients answered all 18 questions, and as we interpreted the results, it was possible to identify their priorities based on their feedback. The patient who buys medicines from the community pharmacy cannot be categorized as a simple client because most of the time this client’s overall picture is influenced by the state of stress caused by his illness. To measure satisfaction, the most important attributes identified were patient loyalty to the pharmacy or to the pharmacist. From the results of the 18 questions, we can rank the most important attributes that lead to an increase in the patient’s degree of satisfaction after being dispensed the medication by the pharmacist. In the first place, 84.22% of patients want a kind pharmacist with communication skills; in the second place, they want a pharmacist who can explain the dosage in detail including side effects or maybe even other additional information. In the next step in the hierarchy, patients appreciate the short waiting time, and the location does not take precedence over loyalty to a particular pharmacist who has provided all the other attributes listed previously. Even if the sales figures lead to successful management, not all patients put a successful brand or low prices of OTC medicines first. Only about 50% of patients choose a pharmacy after promotions or advertisements. What is noteworthy about this study is that of the 30 community pharmacies involved in this study, 27 of them are pharmacies that have marketing with a focus on low prices, advertisements, and commercial offers for OTCs or dietary supplements. In the future, we will focus on a study that also includes pharmacies where the marketing policy is focused on niche pharmaceutical services and not price policies. Although marketing is legally restricted for a community pharmacy, or better said it cannot be subject to the general marketing laws at the level of the country of Romania, the advertising policy includes a lot of focus on the minimum price for OTC drugs and food supplements. However, according to this study, the results confirm that patients need quality pharmaceutical services and subsidiary promotions and prices.

A study that was carried out with great uniformity over the city of Iasi that includes one pharmacy from each district of the city and a response rate of 722 patients within three months is a start for a future approach to pharmaceutical services. We have to keep in mind that in Romania, the first time a pharmaceutical service was implemented was in 2021 [38]. If we were to make a comparison with other countries, the degree of satisfaction and its evaluation would be different because access to these services was different. For example, in Switzerland, the concept of pharmaceutical services was first used in the 19th century [39]. The implementation of quality pharmaceutical services is a positive trend in current pharmaceutical marketing; recognition of the impact of community pharmacists on healthcare value and the need for more optimal medication management suggest that opportunities for community pharmacists to provide patient care may expand in the 21st century [40]. Other countries, where pharmaceutical services were implemented long before they were implemented in Romania, currently have a different approach and a different problem regarding pharmaceutical practice. For example, a study from Scotland indicates that the country wants to prioritize the problems of community pharmacy by addressing six major themes such as promoting the appropriate sale and supply of over-the-counter medicines; patient counseling for prescribed medication; pharmaceutical care to promote medication adherence; promotion and delivery of a Minor Ailment Scheme; pharmaceutical care of vulnerable patients; and effective use of the community pharmacy workforce. Of these, the topic selected as a priority for the next stage of the program was promoting the appropriate sale and supply of over-the-counter medicines [41]. In the case of Romania, the implementation of quality pharmaceutical services can be a solution to increasing the degree of patient satisfaction and implicitly in increasing prevention and patient health. The evaluation of the patient’s degree of satisfaction is considered a predictor of a quality health system. There are many published studies suggesting that satisfied patients are more likely to continue using healthcare services compared to dissatisfied ones [42,43].

### Further Considerations and Study Limitations 

Although labor intensive, distributing the questionnaire to each pharmacy was considered the optimal approach for data collection due to the advanced age of some patients. We know that filling out an online application in an electronic format could be successful, but we wanted to have no criteria for excluding patients. The questionnaire relied on participant self-reporting; therefore, data inaccuracies may result from poor memory or misunderstanding of the questions. There are also limitations in this study because out of the more than 300 community pharmacies in the city of Iasi, only 30 of them agreed to participate in this study [44]. The city of Iasi in Romania has a population of over 270,000 people in the summer months, and during the university term, between October and June, since it is the oldest university center in the whole country, the population exceeds 420,000 people. For this reason, the period we chose for this study was between March and May. Known as the Cultural Capital of Romania, Iași is a symbol of Romanian history. A strong point of this study is that the 30 pharmacies involved in the study covered the entire city area; every important neighborhood in Iasi had a pharmacy involved in the study. Even if they were only 10% of the total pharmacies in the city of Iasi, their distribution was uniform, covering the entire city.

Another limitation of this study was the fact that not all patients wanted to be involved in this project, and only 722 of them had the patience and willingness to complete the questionnaire. Each patient who purchased a drug or dietary supplement from pharmacies was asked if they wanted to be involved in this study and if they would be willing to take the time to complete a questionnaire. Many of them were in a hurry and did not have the time to be involved in this study. In the future, we will build a questionnaire with fewer items that will be easier to complete in a shorter time. 

There are many limitations to this study, but one of the biggest strengths is that it managed to describe a small part of the satisfaction level of patients from the city of Iasi, patients who frequent community pharmacies. Checking the level of patient satisfaction with the pharmacist/pharmacy is a challenge that this study managed to highlight. We define it as a challenge because the evolution of community pharmacies in Romania over the last 30 years has been perceived quite differently. Until the 1990s, all pharmacies were state-owned, there was no concept of a private pharmacy, and implicit access to imported drugs was difficult. After the 1990s in Romania, the pharmaceutical segment could be privatized, and implicitly the pharmacist had his own pharmacy [38]. Later, with the increased consumption of drugs, marketing was oriented toward price and not toward the quality of the pharmaceutical service or the concept of patient-centered medication. In the last 10 years, the community pharmacy in Romania has developed a lot of price advertising campaigns. What we want to emphasize is that as much as the patient is influenced by OTC prices, he is not a typical consumer in the sales area. The pharmacist needs to talk to him, to communicate, and to explain the side effects or dosage of the drugs. In the pharmaceutical field, the product offerings are vast and differ according to multiple parameters, such as active ingredients, the pharmaceutical form, conditioning, bioavailability, efficacy, therapeutical action, or product type (drugs, dietary supplements, medical devices). When talking about product mixes, we must also bring forth the term “product line”, which signifies a homogenous group of products with similar features, such as the same target, the same active ingredient, the same type of promotion, and so on. In 1969, Jerome McCarthy introduced the 4 P (Price, Product, Place, and Promotion) after intense research on the marketing mix. Multiple other specialists have continued their studies and revealed new strategies for successful marketing. Successful marketing means achieving at least the 4 Ps [45,46,47,48]. The marketing mix has been described since the 1960s, but in community pharmacies sometimes you cannot apply all marketing strategies. The patient is not an ordinary consumer; he is a consumer with special needs, and if marketing in the Romanian pharmaceutical area focused more on patient-centered medication and not on standard sales protocols, surely the level of satisfaction with the pharmacy/pharmacist would also increase.

Many of the pharmacists in Romania are convinced that the degree of patient satisfaction is closely related to promotions and prices, but in reality, patient satisfaction is brought about by a multitude of factors. The quality of pharmaceutical services and patient-centered medication can significantly increase the degree of satisfaction and trust in the pharmaceutical act. In the past, another study of 1000 completed questionnaires was also carried out in community pharmacies in Romania, in which it was demonstrated that patients have greater trust in pharmacists when they offer a patient-centered pharmaceutical service [17].

## 5. Conclusions

The degree of patient satisfaction depends on the frequency of visits to the pharmacy, the level of education the patients have, and their social status. The more patients interact with the pharmacist, the higher the satisfaction level. The degree of satisfaction is directly proportional to the level of education and economic stability; the position of the employee can again increase the patient‘s degree of satisfaction. Although many of the pharmacies invest a lot in marketing, pharmacy managers should be more attentive to the needs of patients. Courtesy, more efficient communication, and professionalism are the main needs of patients that can greatly increase the degree of patient satisfaction.

## Figures and Tables

**Table 1:** The characteristics of the patients who were involved in the study.

	Category	Values (No. of Respondents) N = 722
Gender	Male	323
	Female	399
Age	18–35 years	213
	36–55 years	249
	56–65 years	75
	>65 years	185
Status	Employer	370
	Retired	162
	Unemployed	190
Frequency of visits to the pharmacy	Weekly	150
	Monthly	234
	Once every few months	176
	Annual	156

**Table 2:** The questionnaire answered by the patients involved in the study.

The 18 Questions from the Questionnaire Answered by the Patients Involved in the Study	The Stratified Perception of 722 Patients Involved in the Study				
	Strongly Disagree	Disagree	Neutral	Agree	Strongly Agree
1. I appreciate the pharmacist’s kindness.	11	19	70	191	431
2. The level of training of the pharmacist is important to me.	2	30	82	197	411
3. I need my pharmacist to explain to me in detail how to administer the medicines.	18	49	124	202	329
4. I am interested in pharmacy advertisements and promotions.	102	92	169	173	186
5. I want the waiting time for the prescription/purchase of pharmacy products to be short.	54	61	141	177	289
6. I want the pharmacist to inform me about promotional campaigns in the pharmacy.	126	83	176	168	169
7. It is important to me that the pharmacist introduces me to possible side effects or drug interactions.	28	43	133	220	298
8. It would be useful for the pharmacist to carry out my therapeutic scheme.	51	82	171	215	203
9. I appreciate the additional information provided by the pharmacist regarding adverse effects or drug interactions.	38	48	140	179	317
10. I want the pharmacist to be communicative.	31	42	135	218	296
11. It is important to me that the pharmacist has at least 10 years of experience.	181	137	177	134	93
12. The pharmacist’s clothing and neat appearance matter to me.	25	59	145	209	284
13. The recommendation by the pharmacist for medicines with lower prices is useful to me.	48	81	140	177	276
14. I go to pharmacies near my home/workplace.	90	76	140	151	265
15. I choose to go to pharmacies that have a well-known brand (large pharmacy chains).	138	139	187	131	127
16. I select the pharmacy where I go according to the prices charged.	118	81	145	162	216
17. I am interested in the working hours of the pharmacy where I go.	133	89	142	170	188
18. I often choose a pharmacy where I have good communication with the pharmacist.	50	66	144	183	279

## Data Availability

Data are contained within the article.
